# The association of the physical and social environment with mortality in urban areas: an ecological study on the city of Bologna, Italy

**DOI:** 10.1177/14034948241246612

**Published:** 2024-04-25

**Authors:** Teresa Giulia Nicoli Aldini, Despoina Andrioti Bygvraa

**Affiliations:** School of Public Health and Community Medicine, Sahlgrenska Academy, University of Gothenburg, Gothenburg, Sweden

**Keywords:** **U**rban health, built environment, social determinants of health, mortality, ecological study

## Abstract

**Aims:**

The urban environment influences health through many pathways. The aim of the present study was to map the distribution of mortality, environmental predictors (distribution of green areas and transport networks), and social predictors (income deprivation) in the mid-sized city of Bologna (Italy), and to analyse the relationship between these variables.

**Methods:**

The study employed an ecological cross-sectional design using data from public sources. The units of analysis were the 18 city districts. The percentage of green areas, percentage of transport networks and age-standardised mortality rates were calculated for each district. These variables, and an indicator of income deprivation, were plotted on the city map to analyse their distribution. Simple and multiple linear regressions with mortality as the outcome and environmental and social data as predictors were run.

**Results:**

The results showed an unequal distribution of the variables in the city, with the north-eastern districts presenting worse values. Green areas did not result significantly in being related to mortality. The income indicator and transport networks have an impact on mortality in the simple regression, but only transport networks were found to have a statistically significant relation with mortality in the multiple regression.

**Conclusions:**

**The results suggested that living close to major transport networks could affect mortality rates in the city, but further research is needed. Future studies are also needed to analyse the interaction between environmental exposures and socioeconomic factors in affecting health. These findings can be useful for urban planning and health promotion interventions.**

## Background

The environment in which we live affects us daily and in the long term can influence health in many ways. As most of the world’s population now lives in urban settings [1], analysing the effects of the urban environment on health has become a major interest in public and global health for the great potential that it offers for health promotion [2]. Urban planning interventions that adopt a health perspective can influence peoples’ health considerably [3].

Different physical features of cities have impacts on people’s health, by influencing their behaviours and their exposures to hazards or benefits [4]. The presence of trafficked streets and highways, contributing to pollution and temperature increase, has been linked to an increased risk of stroke mortality [5], coronary heart disease mortality [6] and other conditions. Moreover, noise pollution from major roads and railways has been shown to affect mental health and wellbeing [7]. Living near highly trafficked roads can also demote physical activity, and increased motorised transportation networks promote passive transport and a sedentary lifestyle [4]. This is why, according to the World Health Organization (WHO) [1], the phenomenon of urbanisation has played a major role in the high increase in obesity rates and chronic conditions in the past decades. On the other hand, the presence of green and recreational areas incentivises physical and social activities, as well as serving for temperature mitigation and air regulation, which decreases the risk of heat wave-related health episodes and respiratory disease [8]. For these reasons, several studies have linked green areas to various psychological [9] and physical conditions [10].

However, there is still mixed evidence on the influence of different features of the urban environment on health, and the relationship may differ depending on the place, the studied population, as well as other variables [2]. For instance, it is important to account for the socioeconomic inequalities between different parts of a city, because people tend to be clustered based on their socioeconomic characteristics, which may influence their exposure to environmental benefits or hazards and their means to cope with them. This connection between health, environment and social factors, can be analysed through the lens of environmental justice. This concept addresses the complex relationship that intertwines together socioeconomic status, environmental exposures and health. The exposure to environmental resources or hazards tends to be unequally distributed between different socioeconomic groups, for the reason that people with lower socioeconomic status have less political power and voice [11]. This is especially the case for urban settings, where the environment is artificial and is the result of political choices and socioeconomic relations. This unequal distribution results in an amplification of health inequalities between groups, due not only to the differential exposure, but also because lower socioeconomic groups have less means to cope with environmental hazards.

The present study focuses on Bologna, a city of around 400,000 people, situated within the Po Valley, which, due to its geographical morphology, is one of the most polluted areas in Europe [12]. While not hosting heavy industries like other cities of the Po Valley, its central position makes the city a highly important and congested rail and motorway intersection. Moreover, the medieval and compact town structure creates issues with urban vehicle traffic. Vehicle exhausts are, together with residential combustion, the main source of pollution [12], and the city struggles to respect the particulate matter limits set by the European Union (EU) [13].

Despite being a wealthy city, compared with the Italian average, the city presents socioeconomic, health and environmental inequalities, which are reflected in the urban geography. The wealthier southern areas show better health outcomes, such as diabetes prevalence and all-cause mortality, compared with poorer, less educated northern areas [14]. Urban green areas are practically absent in the medieval city centre, sparse in the northern periphery, and more present in the southern, hilly areas [8]. Polluting sources, on the other hand, are more present in the northern areas [15]. The primary cause of death in the city is cardiovascular disease [16]. Like the rest of Italy, the city is experiencing population aging, which poses new health challenges [17].

The environmental issues faced by the city, connected to pollutants and the sparseness of green areas, have raised attention to the challenges that they can pose to citizens’ health. While existing literature has analysed the city environment [8, 12] and the social determinants of health [14], the present study aims to connect the two topics. Bologna presents an interesting case because its medium size and conformation make it similar to other European settings; therefore, this study can help to increase the evidence on the influence of the urban environment on health, and could help planning healthy and sustainable cities.

## Aim

This study aims to investigate the association between environmental and social factors and health in the city of Bologna, Italy. It will map the distribution of different types of land uses, income and mortality, and analyse whether there is an association between these variables. In particular, the following research questions will be considered:

- What are the differences in all-cause mortality between the city districts?
- What is the distribution of income, green areas and transport networks in the city districts?
- Is there an association between green areas distribution and mortality?
- Is there an association between major transport networks distribution and mortality?

## Methods

### Material

The present study is a cross-sectional ecological study. The sample consisted of the 18 districts into which the city of Bologna, Italy, is divided. This is just one of the administrative divisions of the urban area, but it was chosen because it is the smallest division in which mortality data are publicly available.

The data were collected from publicly available sources. The chosen health indicator was the age-standardised mortality rate (ASMR) for each district. ASMRs were calculated by the direct standardisation method. For the calculation, the deaths by age group and the number of people residing in each district for the year 2019 were retrieved from the Office of Vital Statistics of Bologna [18]. The age distribution of the Italian population, serving as a standard to calculate the rates, was retrieved from the National Institute of Statistics [19].

To account for the socioeconomic factor, a variable that had previously been used as a socioeconomic indicator of the area [14, 17] was chosen. This variable, based on data published by the Ministry of Economy [20], is defined as an indicator of socioeconomic deprivation, and was constructed by calculating the percentage of households in the district with a per capita annual income of less than €13,002, which corresponded to 60% of the median income in the city.

The data on green areas and transportation networks in the city of Bologna were retrieved from the 2012 version of the European Urban Atlas (UA), a georeferenced dataset released by the European environmental agency that maps the land uses in European urban areas [21]. This dataset was chosen because it is the most detailed and up-to-date source of this type, and its definition of urban green areas is in line with public health research purposes [22].

The geographical data were managed on the software ArcGIS 10.8. First, they were intersected with the district boundaries, retrieved from the city website [23], to obtain data divided by district. Then, the surface of green areas and of the transport network in each district was computed using the ArcGIS extension Patch Analyst [24]. From this, it was possible to calculate the proportion of surface covered by each land use. The variable of transportation network comprised fast transit roads and railways. The variable ‘green areas’ was created by summing the percentages of the UA labels ‘green urban areas’ and ‘forests’. This choice was made because what UA labels as forests in Bologna are parks that are close to the city and are accessible to the population, therefore they can be considered compatible with the scope of this analysis.

### Analysis

Descriptive statistics were calculated using Stata 17.0 and then plotted on the city map divided by district on ArcGIS, where data were classified in quartiles using Jenks’ natural breaks. This method of classification creates classes with high between-class variance and low within-class variance, providing a realistic view [25].

To assess the association between green areas, transport networks, income and mortality, a number of simple and multiple linear regressions were run. At first, simple linear regressions were done to determine the relation of each predictor with the health outcome. Afterwards, the multiple linear regressions that combined the environmental and the socioeconomic predictors were run. One regression included green areas and the income deprivation indicator as independent variables, the other included the transportation network and the income deprivation indicator as independent variables. Before running the analysis, it was checked that the following assumptions for linear regression were respected: approximate linear relation between predictors and outcome, no multicollinearity between predictors, no heteroskedasticity, normality of residuals and, because this analysis deals with geographically distributed areas, no spatial autocorrelation between residuals. This last assumption was checked with Moran’s I test on ArcGIS.

## Results

In 2019 the total number of deaths in the city were 4701 (districts mean 26,117). The crude mortality rate, that is, the number of deaths for 1000 inhabitants, was 12.02 for the whole city, and it varied from 8.50 to 13.93. Age-standardised rates can be seen in Table I. The mean ASMR was 8.93, (lowest rate 8.38 and highest 9.98).

**Table I.** Crude and age-standardised mortality rates for each district, ordered by percentage of low-income households.

District name	% of households with per capita income <€13,002	Crude mortality rates (CMRs)	Age-standardised mortality rates (ASMRs)
**Mazzini**	21.8	13.93	8.38
**Murri**	22.4	12.54	8.67
**Barca**	22.9	13.90	8.46
**Costa Saragozza**	22.9	11.90	8.57
**Santa Viola**	23.0	10.44	8.93
**Colli**	23.1	10.35	9.07
**Borgo Panigale**	23.2	12.04	8.63
**Corticella**	23.2	12.03	9.18
**San Ruffillo**	23.3	13.32	8.87
**Marconi**	24.3	10.57	8.39
**Lame**	25.5	10.79	8.72
**Saffi**	25.6	12.96	9.64
**San Vitale**	26.4	11.28	9.35
**Galvani**	26.8	10.80	9.46
**Irnerio**	27.7	8.50	8.58
**Malpighi**	28.5	9.66	8.41
**San Donato**	29.9	13.31	9.37
**Bolognina**	30.1	11.49	9.98
**Bologna**	**25.2**	**12.02**	**8.94**

The percentage of green areas in the districts varied from 1.12% to 30.23%, with a mean of 9.55%. The percentage of transport network land use in the districts varied from 0% to 11.71% (mean of 3.27%), with five districts in the southern part of the city presenting a value of 0%. The percentage of people with an income below the threshold varied from 21.8% to 30.1%. See Table II for the summary of descriptive statistics.

**Table II.** Descriptive statistics.

Variable	Mean	Median	Min	Max
Population	21,728.06	19,927.0	8793	38,548
Age-standardised mortality rates	8.92	8.79	8.38	9.98
Income deprivation indicator (%)	25.03	23.8	21.8	30.1
Transport networks (%)	3.27	1.73	0	11.71
Green areas (%)	9.55	5.13	1.12	30.23
Total area (km^2^)	7.82	5.32	0.96	26.12

**Figure 1.**
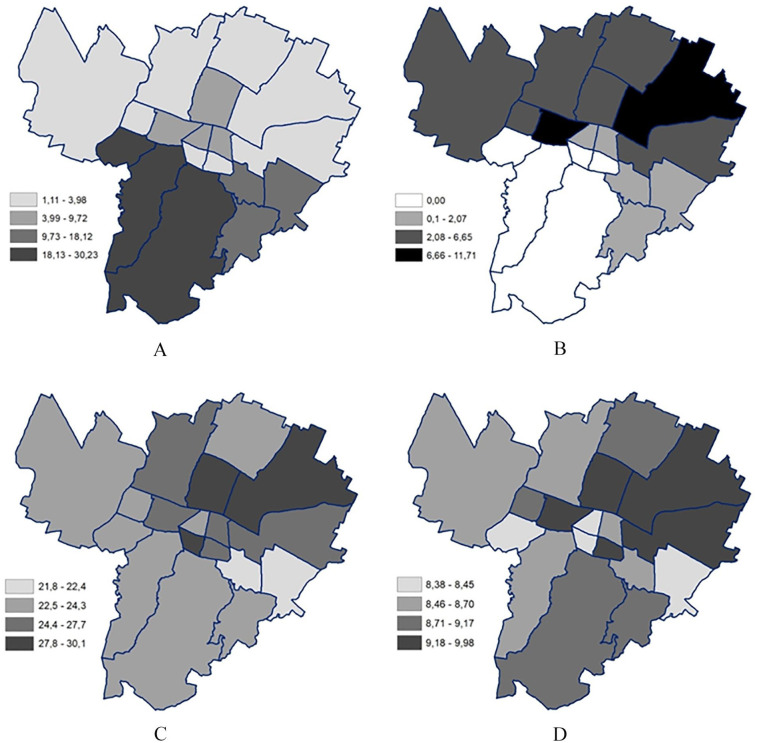
Geographical distribution of the variables divided by district: A. Percentage of green areas in the district; B. Percentage of transport network surface in the district; C. Percentage of low-income households in the district; D. Age-standardised mortality rates, expressed in deaths per 1000.

The plot of the values on the city map showed imbalances in the distribution of the variables (Figure 1). Green areas were more spread in the southern and eastern districts, where they accounted for more than 10% of the land use. On the contrary, major transport networks were absent in the southern areas and more frequent in the northern districts. The income deprivation indicator was more evenly spread throughout the city, but most of the high values were found in the north-eastern areas. Finally, the ASMR showed a cluster of high values in the north-eastern areas. However, values above the mean were also present in some of the southern and western districts.

The simple regressions (Table III) showed an association between ASMR and area income deprivation and between ASMR and transport network, while the association between ASMR and green areas was not significant (*P*=0.23). Both income deprivation and transport networks were positively related to ASMRs. For a one percentage point increase in the number of households with low income, the ASMR increased by 0.093 units (*P*=0.028). For a one percentage point increase in transport networks, the ASMR increased by 0.088 units (*P*=0.004).

**Table III.** Results of the simple linear regressions.

	Age-standardised mortality rates (ASMRs)		
	Coefficient	*P* value	95% CI
Green areas	–0.015	0.230	–0.041–0.011
Transport	0.088	0.004	0.033–0.142
Income deprivation	0.093	0.028	0.011–0.175

CI: confidence interval.

As green areas were non-significant in the simple regression, only the multiple linear regression with transport and income as predictors was considered. Table IV shows the result of this multiple linear regression. The overall model was statistically significant (*F*=7.12, *P*=0.0067). While the predictor ‘transport’ remained significant in this model (*P*=0.023), the income deprivation indicator became non-significant (*P*=0.187). For a one percentage point increase in the presence of transport land use, the ASMR increased by 0.071 units.

**Table IV.** Results of the multiple linear regression.

	Age-standardised mortality rates (ASMRs)		
	Coefficient	*P* value	95% CI
Transport	0.071	0.023	0.011–0.129
Income deprivation	0.051	0.187	–0.028–0.131

CI: confidence interval.

## Discussion

The mapping of the variables showed an unequal distribution of income, green areas and transport networks between the districts. The environmental variables showed a north–south divide, with a higher presence of transport networks and a lower presence of green areas in the northern part. In the case of the socioeconomic indicator, the worse-off areas were in the north, but the better-off areas were found in the two eastern districts. The pattern for ASMR was less clear: the highest ASMRs were still located in the northern areas, but values higher than the mean were also found in some southern and western areas. Overall, the north-eastern areas were the ones to show worse values for all the variables.

The 2020 study by Gentilini et al. [14] also considered the income deprivation indicator and ASMR, but for different years (2011–2015) and at a different level of aggregation. In that case, the worse-off areas were the north-western districts. These differences suggest that adopting a different level of aggregation may have given a different picture of the social and health inequalities in the city. Although mapping of data can help detecting potential patterns, it must be interpreted carefully because methodological choices in the creation of the map influence the final representation [26].

The linear regression showed no association between urban green areas and mortality. The evidence linking urban green areas to health outcomes is mixed [2] and this variability may be due to differences in social and physical contexts between settings [9]. To promote physical activity and other healthy behaviours, green areas have to meet certain characteristics, such as perceived and actual safety, maintenance, accessibility and walkability [27]. These features could not be measured in the present analysis. However, a study [8] has divided Bologna’s green areas into four classes: pocket parks, community parks, neighbourhood parks, and urban parks. The first two classes comprise smaller green areas with reduced relevance for physical activity, while neighbourhood and urban parks indicate big green areas characterised by more vegetation and/or public recreational facilities. To consider the mentioned characteristics, further research could account for this division, as well as other existing indices [28]. Moreover, further studies could analyse the vegetation types present in the green areas, as some types of vegetation may be more beneficial to human health than others [28].

The presence of major transport networks appeared to be related to mortality both in the simple linear regression and in the multiple regression, when the socioeconomic indicator was added to the model. The possible pathway linking the presence of transport networks to mortality is through the exposure to different pollutants which are deemed to increase the risk of chronic diseases [3, 4]. A relationship between the proximity to major roads and mortality has been found in different settings by both ecological studies and individual cohort studies [5–7]. Smaller units of analysis could have given a clearer picture of the exposure to pollutants. However, smaller divisions can increase susceptibility to bias [26].

The release of pollutants is significantly higher within 300 m from the highways [29]; therefore, considering data from the blocks immediately close to the networks may have shown a stronger relationship. Variables and mediators that were not taken into consideration were cause-specific mortality and levels of pollutants. An analysis focusing on pollutants would improve the knowledge of the causal pathway, and it would consider pollutants also released by other sources. Nevertheless, considering the source of pollutants – in this case, transportation networks – can be useful from an urban health perspective in order to orient interventions.

In the simple linear regression, the indicator of income deprivation appeared to be significantly related to mortality. However, the relationship became non-significant when the environmental predictor was added to the model. In the study by Gentilini et al. [14] the same income indicator was significantly correlated with all-cause mortality. However, the time frame and the unit of analysis of the studies differ, and it is possible that the smaller sample used in the present study affected the analysis when a second predictor was added. Another possible statistical explanation of this result would be that the transportation network variable is a mediator in the relationship between the other two variables; therefore, when it is added to the model it covers the previous association [30]. This would be in line with the environmental justice perspective: health hazards such as sources of pollution overlap with areas with a lower socioeconomic status, with the result of widening health inequalities. Further research would be needed to test this hypothesis.

Despite the low generalisability of studies based only on one urban setting, for the reason that every city presents different characteristics [2], the findings from this study can be useful to increase the knowledge on issues of major importance in urban areas – access to green spaces and proximity to major transport networks. Urbanisation is an ongoing process, and knowledge on the effects that the urban environment has on people’s health can help to plan healthier cities.

### Limitations

The present study had some limitations concerning its design. Ecological studies can be subject to ecological bias [31]; therefore, no conclusions on the individual causal path can be drawn. Causation cannot also be determined for the cross-sectional nature of the study, with data collected at a single time point. Moreover, temporality is an issue in this case because in social and spatial epidemiology pathways of causation are likely to have long time lags [32]. However, it should be noted that environmental variables considered in this study were consistent over time, as could be checked by comparing the UA from different years.

Other limitations concern the unit of analysis. The 18 areas are the smallest divisions in which data were publicly available. This sample size might have limited the choice of the number of predictors, because adding more would have compromised the validity of the regression analysis. Moreover, as is often the case for urban health research and spatial epidemiology [32], the units were defined by administrative boundaries, because the socioeconomic and health data are aggregated at that level. However, these boundaries often do not mirror the actual distribution of socioeconomic and health inequalities in the city [33]. It should also be acknowledged that people move around the city, without, of course, being limited by the administrative boundaries, meaning that the environmental predictors affect them in complex ways [32] that could not be considered in this study. Similarly, people migrate from or to different cities, and between different areas of the city, meaning that they experience different degrees of exposure to predictors. As some areas face more turnover than others, this can lead both to over and underestimation of the exposure [26].

Despite the mentioned limitations, this ecological study gives new insights on links between urban environment and health in this area, and generates new hypotheses for future research. Analysing different areas of the same city provides interesting insights into the inequalities between socioeconomic groups within one city and – as urban planning takes place at the city level – can be helpful for intervention purposes [14]. Even small changes in the built environment can have dramatic impacts on both behaviours and exposures, making them a cost-effective public health tool [4]. One strength of this study was to consider both environmental and socioeconomic data. While socioeconomic determinants of health had already been studied in this setting, this was the first study to analyse how the built environment influences health, adding a new perspective on the determinants of health in the city. Combining the analysis of environmental factors with socioeconomic data was necessary for the reason that socioeconomic groups tend to cluster within different areas of the city [26].

## Conclusions

The comparison between the city districts showed differences in all the variables between the northern and the southern areas, and the north-eastern districts appeared to be the ones with worse predictors and outcomes. The regression analysis showed a significant relationship between the presence of transport networks and age-standardised mortality. The presence of green areas was not significantly related to the outcome, while income deprivation was associated in the simple regression, but not when transport networks were added. Even if green areas did not appear to be significant predictors, further research could investigate their role in relation to other health outcomes, including overweight and obesity, hypertension and asthma. Moreover, future research could involve qualitative methods to investigate how people make use of these areas better to assess their effects on physical and mental health.

The significant relationship between the transport network and mortality can serve as hypothesis generating for future studies. Further research could use individual data, different measures of proximity, data on pollutant levels and different health variables. These results add to the already existing evidence that living next to traffic junctions is related to a higher risk of adverse health outcomes. The findings also suggest that health inequalities between social groups may be amplified by different exposures to environmental hazards or resources, as argued by the environmental justice perspective. This relation could be analysed further.

These findings can be useful for urban planning and health promotion interventions, both in the present case and similar urban settings. They add knowledge on the need to reduce polluting sources and the promotion of active and public transportation. The mapping of variables can also be useful to individuate areas of intervention for health promotion.
